# The Distribution of Cardiovascular-Related Comorbidities in Different Adult-Onset Cancers and Related Risk Factors: Analysis of 10 Year Retrospective Data

**DOI:** 10.3389/fcvm.2021.695454

**Published:** 2021-09-14

**Authors:** Qingsong Li, Fei Liu, Yuqi Tang, Sharen Lee, Chao Lang, Lan Bai, Yunlong Xia

**Affiliations:** ^1^Department of Cardiology, The First Affiliated Hospital of Dalian Medical University, Dalian, China; ^2^Faculty of Medicine, The Chinese University of Hong Kong, Hong Kong, SAR China; ^3^Yidu Cloud Technology, Ltd., Beijing, China

**Keywords:** cancer, cardiovascular diseases, risk factors, hypertension, diabetes mellitus

## Abstract

**Introduction:** Understanding the epidemiology of cardiovascular disease (CVD) related comorbidity is a key strategy for improving the outcomes of patients with cancer. Therefore, this study aimed to assess the distribution of cardiovascular comorbidities and cardiovascular risk factors (CVRF) among five cancer sites.

**Methods:** This is a single-centered, cross-sectional study performed in Dalian, China. Between 2008 and 2018, all newly diagnosed cancer in the First Affiliated Hospital of Dalian Medical University, China were screened. Clinical data were extracted from a comprehensive electronic health record system.

**Results:** 35861 patients with lung, colorectal, gastric, breast, and thyroid cancer were collected retrospectively. The most prevalent CVDs in descending order were hypertension (21.9%), followed by coronary heart disease (6.5%), atrial fibrillation (2.9%), and heart failure (1%). The prevalence of hypertension significantly varies between lung (21.3%), colorectal (27.3%), gastric (22.5%), breast (16.7%), and thyroid cancer (22.4%) (*P* < 0.001). CVRF varies with cancer sites. Age, sex, total cholesterol, triglyceride, low-density lipoprotein cholesterol, systolic blood pressure, smoking, alcohol use, and diabetes mellitus (DM) are common risk factors associated with CVD at different cancer sites. The association between DM and presence of CVD was strong in breast (odds ratio [OR] = 4.472, 95% confidence interval [CI]: 3.075–6.504, *P* < 0.001), lung (OR = 3.943; 95% CI: 3.270–4.754, *P* < 0.001), colorectal (OR = 3.049; 95% CI: 2.326–3.996, *P* < 0.001), and gastric (OR = 2.508; 95% CI: 1.927–3.264, *P* < 0.001) cancer.

**Conclusion:** Cancer patients had a significant burden of CVD and increased CVRF. The prevalence of CVRF and CVD comorbidity differ for cancer types. DM remains significantly associated with CVD at different cancer sites except for thyroid cancer.

## Introduction

Cancer is a serious disease that contributes significantly to mortality and morbidity in the global population (1). According to the earlier evidence, nearly half of all cancer deaths are attributed to non-cancer causes, and cardiovascular disease (CVD) is the leading cause of such deaths (2). There are also complex causal relationships between cancer and CVD, and each of them may be caused by, or be a complication of, one or both of the other diseases (3). The significant advancement in cancer management led to a substantial increase in cancer survivors (4). However, this spectacular increase in new cancer therapies (conventional chemotherapy and targeted therapy) is often reported to increase the risk of cardiovascular damage, such as left ventricular dysfunction or heart failure (HF), hypertension (HTN), and arrhythmias (5).

Common biological pathways between CVD and cancer have been described previously. It is well-established that cardiac risk factors have major influences on cancer. The risk intensified in those with diabetes mellitus (DM), dyslipidemia, and obesity (6), suggesting that these cardiovascular risk factors (CVRF) are important features that influence incidence rates of both cancer and CVD. Of note, risk estimation is of crucial importance to recognize cancer patients who are at high risk for CVD; therefore, high-risk patients should be referred to screening for CVD and undergo interventions if indicated to decrease the burden of CVD.

To our knowledge, few studies have reported the prevalence and distribution of CVD among different types of cancer. Prior studies that focused on defining the most common comorbidities in adults with cancer have either used a small sample size or limited data without stratification by cancer type (7–9). A substantial number of cancer and CVD cases are attributable to DM (10–12), whereas the relationship between DM and CVD in cancer patients was rarely reported. Therefore, the goal of this study was to (1) assess the distribution of CVD comorbidities and analyze the associated CVRF among different sites of cancer (lung, colorectal, gastric, breast, and thyroid) and (2) estimate the effect of DM interaction with CVD comorbidities in different cancer sites.

## Methods

### Study Design

This is a single-centered, retrospective cross-sectional study conducted at the First Affiliated Hospital of Dalian Medical University (FAHDMU). Records of patients hospitalized at FAHDMU between 30th May 2008 and 30th September 2018 were retrieved for this study. The statistics department of FAHDMU was contacted to identify the five most common cancer diagnoses at FAHDMU in the last decade. Based on the incidence of respective cancer diagnoses in the database, histologically confirmed lung, colorectal, gastric, breast, and thyroid cancer were included for this study.

### Data Source and Study Population

**Figure 1:**
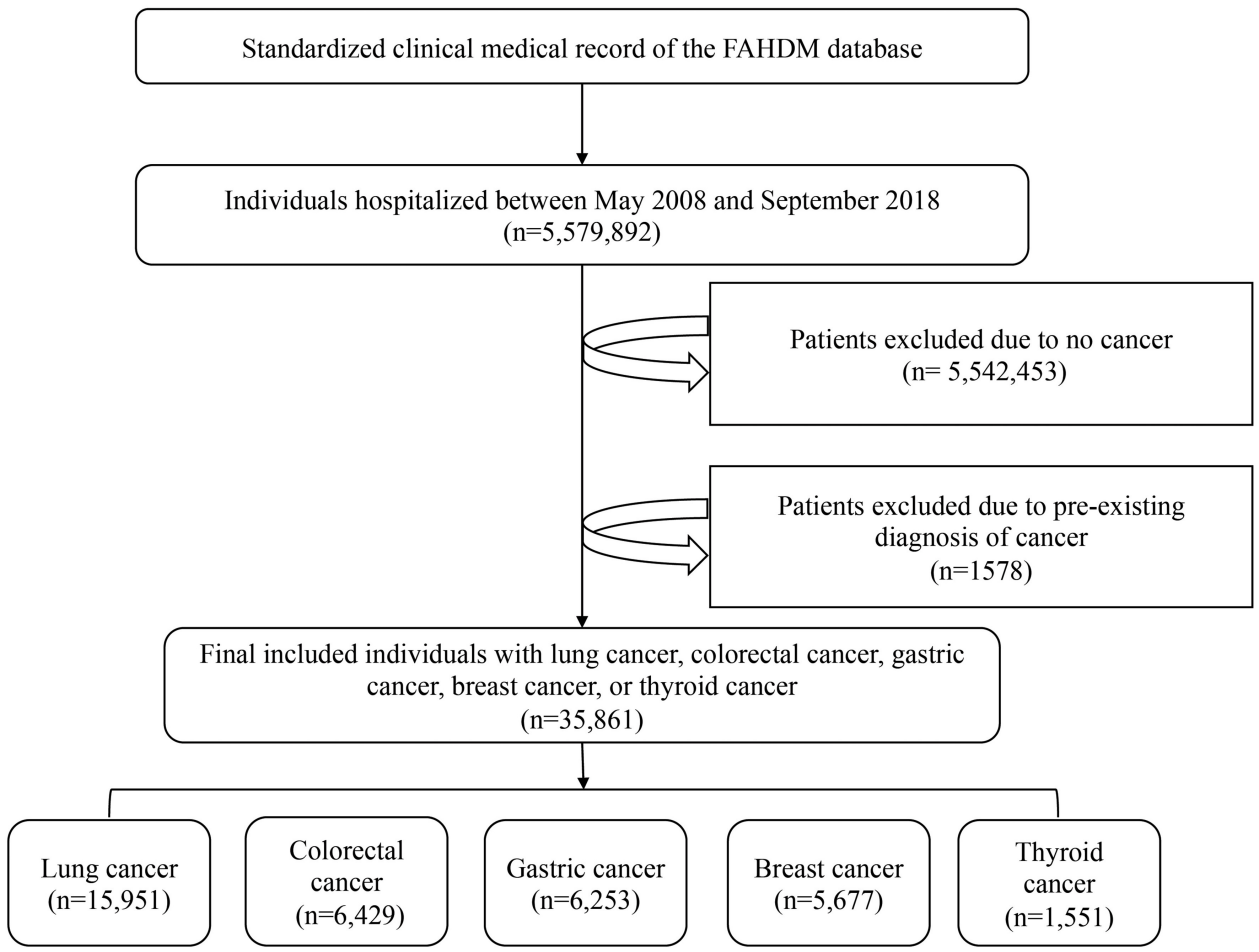
A brief overview of the selection of study participants.

Data for this analysis were obtained from the YiDuloud Electronic Medical Surveillance Network Database (YEMSND). The following patients were excluded from the present study: those without a cancer diagnosis (*n* = 5,542,453) and those with a pre-existing diagnosis of cancer (*n* = 1,578). Finally, a total of 35,861 participants were included in the data analysis. Figure 1 describes a brief overview of the selection of study participants. Institutional review board approval was obtained at FAHDMU. The requirement for informed consent was waived and all procedures listed here were carried out in compliance with Helsinki declaration guidelines.

### Clinical Measurements and Definition of Explanatory Variables

Demographic characteristics, major CVD risk factors, lifestyle-related data, and CVD comorbidities were ascertained from electronic health records by trained health professionals. Clinical measurements, including fasting glucose level, serum concentrations of triglycerides, total cholesterol (TC), high-density lipoprotein cholesterol (HDL-c), and low-density lipoprotein cholesterol (LDL-c) were retrieved. Dyslipidemia was defined as the combination of TC of at least 240 mg/dl, LDL-c of at least 160 mg/dl, and HDL-c of less than 40 mg/dl (13). DM was defined as, fasting blood glucose (FPG) ≥7.0 mmol/L, a self-reported history of DM or current diabetes treatment (14). Smoking was defined as current smoking status or a lifetime consumption of more than 100 cigarettes as described in existing studies (15).

### Definition of Cardiovascular Comorbidity

We considered CVD as co-morbidities when the cancer was first diagnosed during hospitalization. HTN was characterized as systolic blood pressure (SBP) at ≥140 mmHg and/or diastolic blood pressure (DBP) at ≥90 mmHg or a self-reported history of HTN with the current use of antihypertensive drugs. Coronary heart disease (CHD) was defined based on the presence of either angina or coronary artery stenosis of 50% evidenced by medical records (16). HF was defined based on the presence of symptoms of HF and evidence of at least one echocardiographic abnormality (17). Patients were diagnosed with atrial fibrillation (AF) if they met any of the following criteria: (1) AF pattern in baseline 12-lead electrocardiogram (ECG) screening or (2) AF episodes in 24-hour single-lead ECG recording or Holter (18). We defined the first signs of AF during hospitalization as the incident AF cases. Two cardiologists independently performed and read all ECGs to confirm the cases of AF.

### Statistical Analysis

All analyses were performed using SPSS software version 24.0 (SPSS, Chicago, Illinois, USA). Continuous variables were presented using the mean ± standard deviation (SD), whereas categorical data were expressed using frequency (percentage). Statistical significance of differences for categorical variables was tested using χ^2^ (chi-square). Comparisons between continuous data for subgroups with and without CVD were conducted using the independent sample *t*-test. Binary logistic regression analysis was employed to estimate the odds ratio (OR) and 95% confidence interval (CI) for CVD and CVRF associated with the cancers of different sites. The logistic linear regression analysis was adjusted for the targeted CVD type, age, gender, alcohol use, cigarette status, triglycerides, SBP, DBP, HDL-c, LDL-c and DM. Further, we ran a gender-specific analysis to examine the effect of gender on the relationship between CVD types and the presence of cancer at different sites. Statistical significance was defined as *P*-value < 0.05.

## Results

### Baseline Characteristics

Among the 5,579,892 hospitalized patients, a total of 35,861 cancer patients (lung cancer = 15,951, colorectal cancer = 6,429, gastric cancer = 6,253, breast cancer = 5,677, and thyroid cancer = 1,551) were selected in the final analysis. The mean ±SD ages of cancer patients with CVD and without CVD were 69.12 ± 9.89 and 60.30 ± 12.30, respectively. In total, 4,459 of the 18,737 women had CVD, which accounted for 23.8% of the female population whereas 26.6% of the male population had CVD (*P* < 0.001).

The comparison of demographic and clinical variables between cancer patients with and without CVD is presented in Table 1. Overall, 25.15% of the cancer patients were affected by at least one type of CVD. Those with CVD were older than the non-CVD group (*P* < 0.001). Also, those with CVD had a higher mean SBP and DBP than the non-CVD group (*P* < 0.001). The rates of dyslipidemia and DM were significantly higher in the CVD group compared with the non-CVD (*P* < 0.001).

**Table 1:** Baseline characteristics of the participants, (*N* = 35,861).

**Variables**	**No-CVD 26,843 (74.85%)**	**CVD 9,018 (25.15%)**	***P***
Age (years)	60.30 ± 12.30	69.12 ± 9.89	<0.001
Female, *N* (%)	14,278 (53.2%)	4,459 (49.4%)	<0.001
SBP (mmHg)	123.84 ± 20.66	135.04 ± 17.77	<0.001
DBP (mmHg)	76.38 ± 9.664	79.59 ± 10.90	<0.001
LDL (mg/dl)	106.05 ± 30.34	101.25 ± 33.20	<0.001
HDL (mg/dl)	48.07 ± 27.13	44.88 ± 27.25	<0.001
LDL ≥ 160 (mg/dl)	269 (4.1%)	186 (4.4%)	<0.001
HDL <40 (mg/dl)	1,948 (30.0%)	1,694 (40.0%)	<0.001
Triglyceride (mg/dl)	114.80 ± 71.70	124.98 ± 77.87	<0.001
Dyslipidemia (%)	2581 (39.7%)	2,034 (48.0%)	<0.001
Total Cholesterol (mg/dl)	184.89 ± 44.42	175.58 ± 46.55	<0.001
Total Cholesterol ≥240 (mg/dl)	647 (9.9%)	364 (8.6%)	0.019
Smoking N (%)	4,902 (19.0%)	1,910 (21.4%)	<0.001
Alcohol N (%)	2,805 (11.1%)	1,153 (13.2%)	<0.001
Diabetes mellitus, N (%)	1,502 (5.6%)	2,636 (29.2%)	<0.001
Serum uric acid (umol/l)	317.91 ± 100.32	347.71 ± 119.42	<0.000
Hyperuricemia, N (%)	3,207 (13.1%)	1,775 (21.7%)	<0.000
β-blocker use, N (%)	1,100 (4.1%)	2,203 (24.4%)	<0.000
CCB use, N (%)	2,496 (9.3%)	4,102 (45.5%)	<0.000
Diuretic use, N (%)	2,427 (9%)	2,049 (22.7%)	<0.000

*CVD, cardiovascular disease; SBP, Systolic blood pressure; DBP, Diastolic blood pressure; LDL, low-density lipoprotein cholesterol; HDL, high-density lipoprotein cholesterol; CCB, Calcium channel blocker; ACEI, angiotensin-converting enzyme inhibitor; ARB, angiotensin II receptor blocker*.

As shown in Supplementary Table 1, there was a statistically significant difference in the prevalence of smoking between the non-CVD and CVD groups in lung cancer patients (25.7 vs. 29.1%, *P* < 0.001). Similarly, alcohol consumption was significantly higher in the CVD group compared with their counterparts in lung cancer and thyroid cancer patients, but there were no significant differences in the other types of cancers. Interestingly, HDL-c level was lower in the CVD group compared with those without CVD, except for gastric cancer (45.16 vs. 44.93, *P* > 0.05).

### Cardiovascular Diseases Prevalence Among Cancer Patients

**Figure 2:**
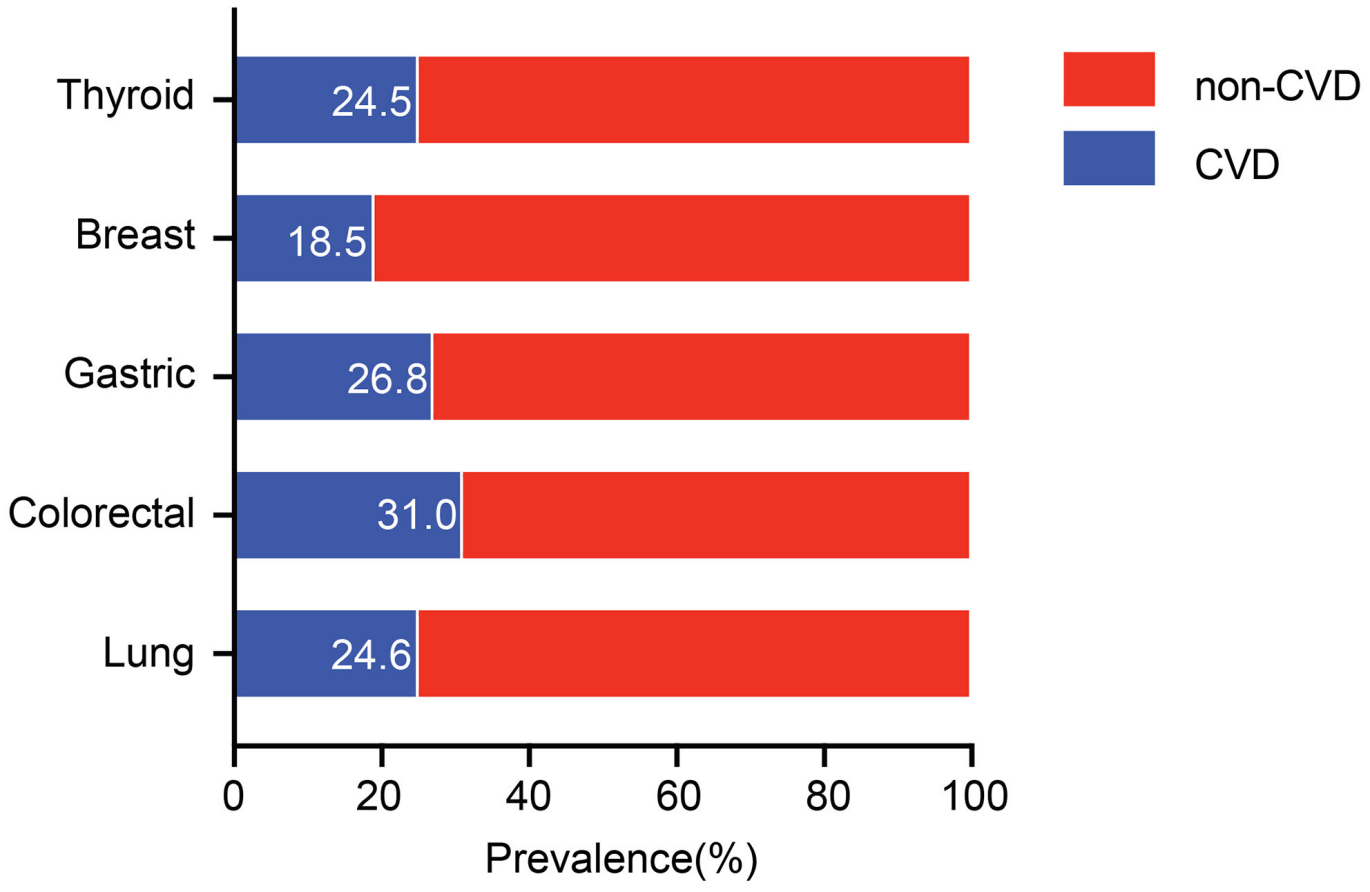
Distribution of CVD among different cancers. CVD, cardiovascular disease.

As shown in Figure 2, the prevalence of comorbid CVD varies between different types of cancer. Patients with colorectal cancer had the highest prevalence of CVD comorbidities (31.0%), followed by patients with gastric (26.8%), lung (24.6%), thyroid (24.5%), and breast (18.5%) cancer.

The most common CVDs in descending order are HTN (21.9%), followed by CHD (6.5%), AF (2.9%), and HF (1%). The prevalence of HTN varies between lung (21.3%), colorectal (27.3%), gastric (22.5%), breast (16.7%), and thyroid cancer (22.4%) with *P* < 0.001.

The highest and the lowest prevalence of CHD was observed among patients with colorectal (7.2%) and breast (4.9%) cancer. The prevalence of AF was the highest in colorectal cancer (4.1%), followed by lung (2.9%) and gastric (2.9%) cancer. The rates of coronary heart disease in lung, colorectal, gastric, and thyroid cancer were 6.6, 7.2, 6.7, and 6.6%, respectively without significant intergroup differences. The distribution of comorbid CVD according to cancer types is presented in Table 2.

**Table 2:** Prevalence of cancer patients with CVDs.

	**All**	**Lung**	**Colorectal**	**Gastric**	**Breast**	**Thyroid**	***P***
	**(*n* = 35,681)**	**(*n* = 15,951)**	**(*n* = 6,429)**	**(*n* = 6,253)**	**(*n* = 5,677)**	**(*n* = 1,551)**	
CVD, N (%)	9,018 (25.1)	3,919 (24.6)	1,996 (31.0)	1,675 (26.8)	1,048 (18.5)	380 (24.5)	<0.001
HTN, N (%)	7,848 (21.9)	3,390 (21.3)	1,755 (27.3)	1,406 (22.5)	950 (16.7)	347 (22.4)	<0.001
CHD, N (%)	2,320 (6.5)	1,054 (6.6)	461 (7.2)	421 (6.7)	281 (4.9)	103 (6.6)	<0.001
AF, N (%)	1,041 (2.9)	470 (2.9)	263 (4.1)	182 (2.9)	93 (1.6)	33 (2.1)	<0.001
HF, N (%)	345 (1.0)	160 (1.0)	76 (1.2)	54 (0.9)	45 (0.8)	10 (0.6)	0.110

*HTN, hypertension; AF, atrial fibrillation; CHD, coronary heart disease; HF, heart failure*.

### Sex-Based Comparison of Cardiovascular Disease Comorbidity

**Figure 3:**
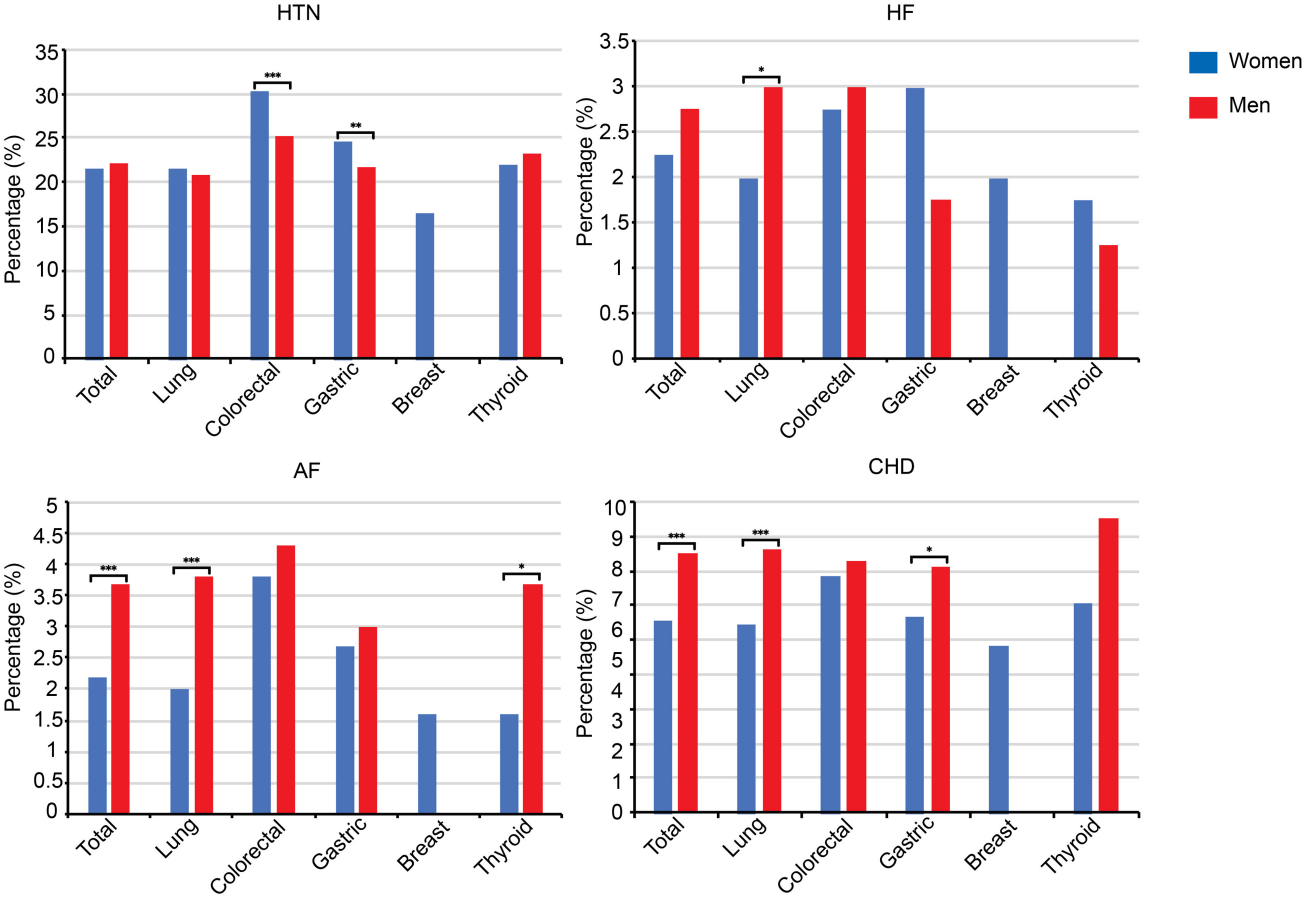
Comparison of CVD comorbidities between men and women according to cancer categories. HTN, hypertension; HF, heart failure; AF, atrial fibrillation; CHD, coronary heart disease; *P < 0.05, ***P < 0.001.

The overall rate of cancer patients with CVD was significantly higher amongst male patients (*P* < 0.001). Likewise, male patients with colorectal and gastric cancer had a higher prevalence of HTN comorbidities than their female counterparts (*P* < 0.001). Similarly, the proportion of CHD and AF in lung cancer patients was significantly higher in men in comparison to women (*P* < 0.01). Also, there was a statistically significant difference in AF rate between men and women with thyroid cancer (1.6 vs. 3.7%, *P* < 0.05). More detailed information on the gender differences in comorbid CVD according to cancer categories is presented in Figure 3.

### Risk Factors of CVD in Selected Cancer

We evaluated the risk factors associated with CVD among patients stratified by cancer categories (Table 3). The findings of the present study demonstrated a positive association between DM and the presence of CVD in all studied cancer except for thyroid cancer. The association between DM and presence of CVD was strong in breast (OR = 4.472, 95% CI: 3.075–6.504, *P* < 0.001), lung (OR = 3.943; 95% CI: 3.270–4.754, *P* < 0.001), colorectal (OR = 3.049; 95% CI: 2.326–3.996, *P* < 0.001), and gastric (OR = 2.508; 95% CI: 1.927–3.264, *P* < 0.001) cancer.

**Table 3:** Cardiovascular risk factors among the different cancer sites.

**Cancer type**		**B**	**S.E**.	**Wald**	**OR**	**95% C.I**.	***P***
Lung	Female	−0.163	0.075	4.648	0.85	0.733–0.985	0.031
	Age	0.058	0.004	265.102	1.06	1.052–1.067	<0.001
	Tch	−0.004	0.001	22.268	0.996	0.994–0.998	<0.001
	TG	0.002	0.001	16.984	1.002	1.001–1.003	<0.001
	SBP	0.034	0.002	234.633	1.035	1.030–1.039	<0.001
	DM	1.372	0.095	206.362	3.943	3.270–4.754	<0.001
Colorectal	Age	0.052	0.006	87.841	1.054	1.042–1.065	<0.001
	Tch	−0.007	0.001	26.195	0.993	0.990–0.996	<0.001
	SBP	0.039	0.004	109.177	1.04	1.032–1.048	<0.001
	DM	1.115	0.138	65.249	3.049	2.326–3.996	<0.001
	Female	−0.33	0.132	6.238	0.719	0.555–0.931	0.013
	Smoke	0.651	0.198	10.792	1.917	1.300–2.827	<0.001
	Alcohol	−0.51	0.218	5.452	0.601	0.392–0.921	0.02
Gastric	Age	0.048	0.005	91.369	1.049	1.039–1.059	<0.001
	TG	0.005	0.001	29.165	1.005	1.003–1.007	<0.001
	SBP	0.039	0.003	141.811	1.04	1.033–1.047	<0.001
	DM	0.919	0.134	46.817	2.508	1.927–3.264	<0.001
	Tch	−0.009	0.001	44.989	0.991	0.989–0.994	<0.001
Breast	Age	0.089	0.007	154.198	1.094	1.078–1.109	<0.001
	Tch	−0.023	0.005	21.172	0.977	0.968–0.987	<0.001
	TG	0.002	0.001	6.517	1.002	1.001–1.004	<0.001
	DM	1.498	0.191	61.462	4.472	3.075–6.504	<0.001
	LDL	0.024	0.007	11.809	1.024	1.010–1.038	<0.001
	DBP	0.015	0.007	4.384	1.015	1.001–1.029	0.036
Thyroid	Age	0.081	0.012	42.353	1.085	1.058–1.112	0.001
	HDL	−0.008	0.005	2.184	0.992	0.982–1.003	0.139
	TG	0.005	0.002	10.691	1.005	1.002–1.008	0.001
	SBP	0.046	0.009	26.888	1.047	1.029–1.066	0.001
	Tch	−0.008	0.003	5.379	0.992	0.986–0.999	0.020

*SBP, systolic blood pressure; DBP, diastolic blood pressure; LDL, low-density lipoprotein cholesterol; HDL, high-density lipoprotein cholesterol; Tch, total cholesterol; TG, triglyceride; SE, standard error; OR, odds ratio; CI, confidence interval. Adjusted for age, gender, cigarette status, alcohol use, triglycerides, SBP, DBP, HDL and LDL*.

In the multivariate model, the logistic regression analysis presented that old age, high levels of triglycerides, and increased levels of SBP were independent risk factors for lung, colorectal, and gastric cancer. We also observed smoking (OR = 1.917; 95% CI: 1.300–2.827, *P* < 0.001) was independently associated with colorectal cancer. Similarly, breast cancer patients with increased DBP had higher rates of CVD. Likewise, being female was associated with a lower prevalence of CVD in lung (OR = 0.850; 95% CI = 0.733–0.985, *P* < 0.05) and colorectal (OR = 0.719, 95% CI 0.555–0.931, *P* < 0.05) cancer.

## Discussion

There are three major findings to the present study: (1) cancer patients carry a significant burden of CVD-related comorbidities; (2) the prevalence of selected CVD (HTN, AF, HF, and CHD) varies by cancer type; (3) HTN is the most common CVD comorbidity among all studied cancers while DM is the most common CVRF.

The present study showed that cancer patients carry a high CVD burden. According to a recent multicentered study, 18.0% of the cohort were affected by at least one type of CVD with a mean age of 55 years at cancer diagnosis (8). The relatively high prevalence of comorbidity (25.15%) in our study may be related to the older mean age of 69.12 ± 9.89 at diagnosis. It is supported by a study by Giovannucci et al. (19), which found that 78% of new cancers were related to aging (≥55 years old). We also focused on a limited number of CVD comorbidity, which could also contribute to the difference in the prevalence of CVD.

In this study, cancer patients of lung, colorectal, gastric, breast, and thyroid suffered from a high prevalence of CVD. We found that among the studied cancers, colorectal cancer had the highest prevalence of cardiovascular comorbidities. Colorectal cancer is the third most commonly diagnosed cancer around the world with a male predominance (20). Given the prevalence of colorectal cancer and CVD, in addition to the increased CVD risk in males, it is not surprising to find a high coexistence between CVD and colorectal cancer.

Also, results from the current study demonstrated that colorectal cancer patients diagnosed with DM had an increased risk for HTN, AF, and CHD. It has been previously demonstrated that HTN increases the risk of colorectal cancer (21, 22). Considering the strong relationship between HTN and other CVD such as AF and CHD, as well as the strong inter-relationship between HTN and DM, it is important to keep in mind the increased CVD risk in the management of cancer patients. A metanalysis by Zhu et al. indicated that DM patients will have five years shorter in their survival from the colorectal, colon, and rectal cancers compared to patients without DM (23). Hence, our result indicates that a holistic control strategy of CVRF is not inferior to a DM control strategy in preventing the risk of CVD in cancer patients.

HTN is the most common form of CVD and it is linked to other major CVD complications (24). In our study, the distribution of HTN was significantly higher than that of other cardiovascular comorbidities in the studied cancers. Recent meta-analyses of observational studies have reported higher risks for colorectal, prostate, and breast cancer in hypertensive compared with normotensive individuals (25–27). However, the association between HTN and cancer is still not clear. It is hypothesized that the two conditions share common risk factors and mechanisms of pathogenesis. For instance, it has been speculated that predisposition to cancer is aggravated by chronic inflammation (28) and lipid peroxidation (29). Also, another possible biological explanation that links HTN and cancer may be via angiogenic factors. Hypertensive subjects have been found to have an increased concentration of serum vascular endothelial growth factor (VEGF), a hormone that plays a critical role in the promotion of angiogenesis in tumors (30). Further, experimental studies have implicated a potential role of the renin-angiotensin-aldosterone system (31). Recently, blockade of the angiotensin II type 1 receptor in mice was found to attenuate the growth and metastatic potential of renal cell carcinoma (32).

In general, CVD and cancer are viewed as two distinct disease entities. However, CVD and cancer interact on multiple levels, sharing common causal mechanisms and epidemiologic risk factors (3). The findings of the present study demonstrated a positive association between DM and the presence of CVD in all studied cancer except for thyroid cancer. It has been previously demonstrated that type 2 DM patients embrace a significantly higher risk of cancer (33). It is uncertain whether the relationship between DM and cancer is direct or indirect given the common risk factors Earlier evidence documented that the duration of DM is an important factor in the progress of cancer among insulin-using type 2 DM patients (34). However, the pathogenic mechanisms remain unclear. Given the burden of CVD and cancer tends to increase in advanced age groups, the combination of the duration of DM along with increased age may confound the association between DM and cancer. The mechanisms of such increased cancer risk in diabetic patients may be linked with hyperinsulinemia, increased insulin resistance, inflammation, and oxidative stress (35).

### Strength and Limitation

The major advantage is the relatively large sample size. Furthermore, blood pressure reading was measured by trained health professionals and was not self-reported to ensure accurate measurements. Detailed data on lifestyle, blood pressure measurements, lipid panel, and medical history were also available, allowing adjustment for potential confounders. However, the present study has several limitations. First of all, the blood pressure and lipid panel were measured only at a one-time point. This was a single-center cross-sectional study and was subject to inherent limitations associated with cross-sectional data, such as the inability to establish cause-effect relationships. Also, the present study is a single-centered study in China, which can limit its generalizability given the effect of lifestyle and ethnicity on CVD risk. Consequently, the results of the present study should be validated by cohorts from other countries. Moreover, the lack of continuous ECG monitoring means that patients with paroxysmal AF may remain undetected and the lack of non-cancer group lead to be impossible to compare the distribution of cardiovascular comorbidities between cancer and non-cancer participants.

## Conclusion

In conclusion, this cross-sectional study confirmed that cancer patients had a significant burden of CVD and increased CVRF. Prevalence of CVRF and CVD comorbidity differ for cancer type. Among the studied cancers, colorectal cancer had the highest prevalence of cardiovascular comorbid conditions. DM remained a major risk factor for CVD comorbidity at different cancer sites except for thyroid cancer. Medical practitioners should enhance the delivery of high-quality cardiovascular care for patients with cancer to control their cardiovascular risk and provide routine cardiovascular monitoring.

## Data Availability Statement

The original contributions presented in the study are included in the article/Supplementary Materials, further inquiries can be directed to the corresponding author/s.

## Ethics Statement

Written informed consent was obtained from the individual(s) for the publication of any potentially identifiable images or data included in this article.

## Author Contributions

YX designed this study. FL and YT were in charge of data analysis and data collection. QL drafted the article. FL and SL did the critical revision of article. LB and CL conducted the data collection. All authors have read and approved the final manuscript.

## Funding

This work was supported by the National Natural Science Foundation of China (Grant Number 81970286) and the Natural Science Foundation of Liaoning Province (2019-ZD-0644).

## Conflict of Interest

CL and LB was employed by the company Yidu Cloud Technology, Ltd., Beijing, China. The remaining authors declare that the research was conducted in the absence of any commercial or financial relationships that could be construed as a potential conflict of interest.

## Publisher's Note

All claims expressed in this article are solely those of the authors and do not necessarily represent those of their affiliated organizations, or those of the publisher, the editors and the reviewers. Any product that may be evaluated in this article, or claim that may be made by its manufacturer, is not guaranteed or endorsed by the publisher.
