# Supplementary material for: The Distribution of Cardiovascular-Related Comorbidities in Different Adult-Onset Cancers and Related Risk Factors: Analysis of 10 Year Retrospective Data
Source: Front Cardiovasc Med. 2021 Sep 14;8:695454. doi: 10.3389/fcvm.2021.695454 (PMC8476781; doi:10.3389/fcvm.2021.695454)
Supplement: Supplementary file 1 [file Table_1.DOCX]

**Table S1. Baseline characteristics of the participants by cancer type**

| **Variables** | **Lung cancer (n=15951)** | | | **Colorectal cancer (n=6429)** | | | **Gastric cancer (n=6253)** | | | | **Breast cancer (n=5677)** | | | **Thyroid cancer (n=1551)** | | |
| --- | --- | --- | --- | --- | --- | --- | --- | --- | --- | --- | --- | --- | --- | --- | --- | --- |
|  | No-CVD  12032 (75.43%) | CVD  3919 (24.56%) | ***P*** | No-CVD 4433 (68.95%) | CVD  1996 (31.08%) | ***P*** | No-CVD 4578 (73.21%) | CVD  1675 (26.79%) | ***P*** | No-CVD  4629 (81.54%) | | CVD  1048 (18.46%) | ***P*** | No-CVD 1171 (75.50%) | CVD  380  (24.50%) | ***P*** |
|  |  |  |  |  |  |  |  |  |  |  | |  |  |  |  |  |
| Age (years) | 61.47±11.16 | 68.95±9.62 | ＜0.001 | 63.69±12.23 | 71.29±9.62 | ＜0.001 | 63.52±11.56 | 70.24±9.38 | ＜0.001 | 54.08±11.46 | | 66.55±9.98 | ＜0.001 | 47.60±13.00 | 61.76±10.86 | ＜0.001 |
| Female, N (%) | 5766(47.9%) | 1794(45.8%) | 0.019 | 1722(38.8%) | 840(42.1%) | 0.014 | 1295(28.3%) | 502(30.0%) | 0.193 | 4604(99.5%) | | 1041(99.3%) | 0.618 | 891(76.1%) | 282(74.2%) | 0.459 |
| SBP (mmHg) | 124.83±19.21 | 135.85±18.43 | ＜0.001 | 124.54±22.14 | 135.08±17.14 | ＜0.001 | 121.05±15.00 | 132.15±17.02 | ＜0.001 | 124.10±27.66 | | 136.74±17.60 | ＜0.001 | 120.68±14.42 | 134.69±16.44 | ＜0.001 |
| DBP (mmHg) | 76.66±9.85 | 79.83±11.13 | ＜0.001 | 76.48±9.62 | 79.71±10.48 | ＜0.001 | 74.98±9.65 | 78.12±10.81 | ＜0.001 | 77.10±9.16 | | 80.44±10.89 | ＜0.001 | 75.85±9.35 | 80.68±10.58 | ＜0.001 |
| Tch (mg/dl) | 185.56±43.30 | 178.94±46.28 | ＜0.001 | 183.00±44.42 | 170.67±43.50 | ＜0.001 | 99.50±29.37 | 92.76±34.10 | ＜0.001 | 201.90±44.53 | | 191.60±48.37 | ＜0.001 | 191.33±43.66 | 185.49±40.61 | 0.156 |
| LDL (mg/dl) | 107.35±29.73 | 103.87±33.34 | ＜0.001 | 106.00±31.00 | 98.53±30.72 | ＜0.001 | 173.50±43.01 | 161.63±46.22 | ＜0.001 | 112.27±31.48 | | 109.03±33.38 | 0.083 | 107.82±28.86 | 105.27±29.16 | 0.369 |
| HDL (mg/dl) | 48.02±21.82 | 44.81±12.60 | ＜0.001 | 47.67±38.12 | 43.04±22.37 | 0.002 | 45.16±31.95 | 44.93±52.61 | 0.895 | 52.43±12.43 | | 47.61±12.10 | ＜0.001 | 52.51±35.08 | 46.45±11.72 | 0.015 |
| TG (mg/dl) | 113.99±70.36 | 123.76±75.86 | ＜0.001 | 116.72±69.48 | 118.68±72.66 | 0.558 | 103.44±58.11 | 115.81±73.15 | ＜0.001 | 130.04±86.19 | | 145.95±85.45 | 0.001 | 131.19±99.62 | 151.77±99.27 | 0.035 |
| Dyslipidemia, % | 1133(39.7%) | 893(46.0%) | ＜0.001 | 405(39.8%) | 388(49.2%) | ＜0.001 | 688(45.8%) | 474(56.0%) | ＜0.001 | 292(31.3%) | | 189(43.6%) | ＜0.001 | 63(32.1%) | 90(40.0%) | 0.095 |
| Tch≥240 mg/dl | 286(10.0%) | 183(9.4%) | 0.499 | 88(8.6%) | 52(6.6%) | 0.108 | 94(6.3%) | 40(4.7%) | 0.124 | 157(16.8%) | | 67(15.5%) | 0.524 | 22(11.2%) | 22(9.8%) | 0.628 |
| LDL≥160 mg/dl | 128(4.5%) | 97(5.0%) | 0.409 | 35(3.4%) | 26(3.3%) | 0.872 | 40(2.7%) | 23(2.7%) | 0.938 | 59(6.3%) | | 31(7.2%) | 0.566 | 7(3.6%) | 9(4.0%) | 0.819 |
| HDL＜40 mg/dl | 847(29.7%) | 725(37.3%) | ＜0.001 | 324(31.8%) | 343(43.5%) | ＜0.001 | 595(39.6%) | 432(51.0%) | ＜0.001 | 139(14.9%) | | 124(28.6%) | ＜0.001 | 43(21.9%) | 70(31.1%) | 0.034 |
| Smoking N (%) | 2990(25.7%) | 1130(29.1%) | ＜0.001 | 742(17.4%) | 352(17.9%) | 0.597 | 1055(24.0%) | 387(23.5%) | 0.636 | 42(0.9%) | | 9(0.9%) | 0.817 | 73(6.5%) | 32(8.5%) | 0.192 |
| Alcohol N (%) | 1463(13.0%) | 597(15.8%) | ＜0.001 | 544(12.9%) | 236(12.2%) | 0.421 | 733(17.1%) | 289(17.7%) | 0.562 | 26(0.6%) | | 8(0.8%) | 0.493 | 39(3.5%) | 23(6.1%) | 0.027 |
| DM, N (%) | 587(4.9%) | 1094(27.9%) | ＜0.001 | 375(8.5%) | 650(32.6%) | ＜0.001 | 327(7.1%) | 439(26.2%) | ＜0.001 | 156(3.4%) | | 340(32.4%) | ＜0.001 | 57(4.9%) | 113(29.7%) | ＜0.001 |

**Abbreviations**: CVD, cardiovascular disease; SBP, Systolic blood pressure; DBP, Diastolic blood pressure; Tch, total cholesterol; LDL, low-density lipoprotein cholesterol; HDL, high-density lipoprotein cholesterol; TG, triglyceride; DM, diabetes mellitus.
